# Salivary Oxytocin as a Biomarker in Psychedelic Assisted Psychotherapy

**DOI:** 10.1192/j.eurpsy.2025.2110

**Published:** 2025-08-26

**Authors:** L. Cazorla, S. Alaux, C. Mabilais, C. Amberger, A. Buchard, G. Thorens, P. Louise, Z. Daniele, T. Aboulafia Brakha

**Affiliations:** 1 Psychiatry, Geneva University Hospitals, Geneva, Switzerland

## Abstract

**Introduction:**

One of the main mechanisms of action of LSD in psychedelic-assisted psychotherapy (PAP) is the activation of 5HT2A receptors. This triggers a cascade of neurochemical processes with the release of neurotransmitters and neurohormonal substances. The release of oxytocin in this process is well documented in pre-clinical studies, with recent evidence in healthy subjects. We have no data on oxytocin reactivity during LSD treatment in patients with psychiatric problems. It would be of great scientific interest to identify treatment biomarkers in this field.

**Objectives:**

The main objective of this pilot study is to obtain preliminary data on the reactivity of salivary oxytocin during a single LSD intake as part of PAP for anxiety disorders or depression. The secondary objective is to establish preliminary correlations between variations in oxytocin levels, intensity of perceived effects, intensity of psychedelic mystical experience, and the overall clinical response to treatment (anxiety-depression symptoms).

**Methods:**

Participants are recruited among patients with resistant anxiety-depressive disorders enrolled in the psychedelic-assisted psychotherapy (PAP) program of the Division of Addictology, Department of Psychiatry (Geneva University Hospital). Salivary oxytocin is measured at four different time-points: before LSD intake, then 60, 90 and 180 minutes after intake. Self-perceived intensity of effects is also measured at these time points and a self-report questionnaire of mystical experience (MEQ-30) is administered at the end of the LSD session. Of note, self-reported symptoms of depression (BDI-II) and anxiety (SATI-T) are measured when participants enroll the PAP program, then a few months later right before the treatment and three weeks after treatment. ClinicalTrials.gov:NCT06557239

**Results:**

Six participants (out of 10 planned) have completed the entire protocol. Four additional participants will receive treatment within the next two weeks. Saliva samples are stored at -20.C and will be sent for analysis once recruitment will be over (November 2024). A significant effect of time (P= 0.02) of perceived intensity effects is observed across different time-points (30, 60, 90, 180, 360 minutes after treatment), with a peak effect at 180 minutes. We also observe a significant interaction (P=0.03) between self-perceived intensity of effects and the intensity of mystical experience measured with the MEQ-30. BECK and STAI-T scores will be analysed after post-treatment assessment.

**Conclusions:**

Our preliminary data show a clear effect of self-perceived treatment intensity and a relation between this effect and self-reported mystical experience. It will be of great interest to include oxytocin data in this analysis as well as the evolution of self-reported symptoms of anxiety and depression. With the successful recruitment and easy adherence to the protocol, we will certainly have all data available by the end of 2024.

**Disclosure of Interest:**

None Declared

